# The Supertree Tool Kit

**DOI:** 10.1186/1756-0500-3-95

**Published:** 2010-04-08

**Authors:** Katie E Davis, Jon Hill

**Affiliations:** 1 Faculty of Biomedical & Life Sciences, Division of Ecology & Evolutionary Biology, Graham Kerr Building, University of Glasgow, Glasgow, G12 8QQ, UK; 2 Applied Modelling and Computation Group, Earth Science and Engineering, Imperial College London, London, SW7 2AZ, UK

## Abstract

**Background:**

Large phylogenies are crucial for many areas of biological research. One method of creating such large phylogenies is the supertree method, but creating supertrees containing thousands of taxa, and hence providing a comprehensive phylogeny, requires hundred or even thousands of source input trees. Managing and processing these data in a systematic and error-free manner is challenging and will become even more so as supertrees contain ever increasing numbers of taxa. Protocols for processing input source phylogenies have been proposed to ensure data quality, but no robust software implementations of these protocols as yet exist.

**Findings:**

The aim of the Supertree Tool Kit (STK) is to aid in the collection, storage and processing of input source trees for use in supertree analysis. It is therefore invaluable when creating supertrees containing thousands of taxa and hundreds of source trees. The STK is a Perl module with executable scripts to carry out various steps in the processing protocols. In order to aid processing we have added meta-data, via XML, to each tree which contains information such as the bibliographic source information for the tree and how the data were derived, for instance the character data used to carry out the original analysis. These data are essential parts of previously proposed protocols.

**Conclusions:**

The STK is a bioinformatics tool designed to make it easier to process source phylogenies for inclusion in supertree analysis from hundreds or thousands of input source trees, whilst reducing potential errors and enabling easy sharing of such datasets. It has been successfully used to create the largest known supertree to date containing over 5000 taxa from over 700 source phylogenies.

## Findings

### Background

A supertree is defined as an estimate of phylogeny assembled from smaller phylogenies. These partial phylogenies (or source trees) must have some taxa in common, but not necessarily all [1]. Modern supertrees can contain hundreds or thousands of taxa and are constructed from hundreds of source phylogenies requiring the collection of large amounts of data [2-4]. These phylogenies are of great use in, for example, comparative biology [5], and macroevolutionary studies [6]. Source phylogenies for supertree construction are typically stored in NEXUS files [7], which contain the phylogenetic relationships between the taxa included in the source study. Collecting, storing and processing this volume of data becomes an arduous and error-prone task as the number of NEXUS files increases. One cannot simply collect a few hundred source phylogenies and use these as published to create a supertree. Criticisms of supertrees have arisen for a variety of reasons, both practical and philosophical. Data quality is the main practical issue [8] and is the main consideration of the Supertree Tool Kit, as the results from any supertree analysis can only be as good as the input data [9]. In order to minimise potential sources of bias on supertree construction a clear, rigorous, transparent method of processing and assessing the source phylogenies is required.

Previous work in this area has proposed two protocols to carry out this processing [2,9]. The Supertree Tool Kit (STK) is designed to implement the latter protocol of Davis [2], based on that of Bininda-Emonds *et al. *[9] and has been used successfully in the construction of two large supertrees at this time, containing 436 [3] and 5276 taxa [2]. The protocol implemented briefly consists of the following steps:

1. **Data collection and entry: **Collecting data from the literature and encoding printed trees as NEXUS-formatted tree files. Meta-data about the source phylogeny is stored in a corresponding XML file.

2. **Standardisation of terminal taxa: **Removal of synonyms, vernacular names and subspecies. Standardisation of taxonomic level.

3. **Source tree independence: **Remove redundancy within the dataset that would otherwise unfairly up-weight data. Here, non-independence is defined as two or more studies that use the same character data and have identical taxa or two or more studies that use the same character data and where one taxa set is a subset of the other. This is not a standard definition of independence and has no statistical meaning, but is the only practicable definition that can be applied to supertree studies. It is important to recognise that according to other definitions there may be some issues of data overlap remaining even if the above conditions are met.

4. **Check adequate taxonomic overlap of source trees: **Ensure that there is adequate taxonomic overlap between source phylogenies, in other words all source phylogenies must fulfill the basic requirement for supertree construction of each source (rooted) phylogeny has at least two taxa in common with one other (rooted) phylogeny [1]

5. **Matrix creation: **Create a matrix suitable for analysis in PAUP* [10], TNT [11] or similar, if required.

### Previous work

There are no published software tools of this kind - the STK is unique in this respect. Previous work has been on ad-hoc scripts that automate a single part of suggested protocols, for example "synonoTree" [9], or "SuperMR" [12]. However, these are one-offs, which do not use standard NEXUS parsers for Perl and are not integrated into a common framework. Of course, these two scripts alone cannot be used to implement the whole protocol as proposed by Davis [2] or Bininda-Emonds *et al. *[9] and as such represent a partial implementation at best. Some tools that aid with the storage of NEXUS files do exist, such as TreeBASE [13] which stores a large range of phylogenies alongside the meta-data containing information on how the tree was originally constructed. However, this meta-information is not readily available for download.

### Implementation

The STK is written in Perl and consists of a Perl module and a number of individual scripts. The main requirements of the STK are Bio::NEXUS, a Perl API for handling NEXUS files [14] and Perl (version 5 or greater), with the addition of a few standard Perl modules. All scripts are run from the command line. The executables use the functionality of the Perl module and implement the protocol outlined above. The Perl module can be used as the basis for creating further executables.

#### Data Storage

The STK uses three data formats: NEXUS [7], XML and taxa substitution files. The minimum data input for the STK is a collection of NEXUS-formatted tree files [7]. These contain the taxa and the phylogenetic information that is a minimum requirement for source data. However, these files miss some crucial information that is required to fully implement the data processing protocols outlined above; namely the bibliographic and analytical information for each source tree. In order to store these data, we use the second type of file: XML files, which are a meta-data storage medium. XML defines data using "tags" and "attributes". The STK XML format contains several such tags. The XML files implemented by the STK contain metadata about each source tree, such as bibliographic information, i.e. authors, journal, year etc., included taxa, and characters used and the algorithmic method used to construct the source tree. This format was chosen, rather than simply creating a document in Word or Excel as has been done in some previous supertree studies e.g. [15], as it is very easy to extract trees required for any specific analysis, i.e. morphological or molecular data only by parsing the XML. In addition, the combination of two files to store the data enables "data integrity" checks to be carried out throughout processing, which ensures that the source data and associated meta-data stay in step during processing. The XML files are also an essential part of some aspects of the data processing protocol. Checking data independence, for example, requires knowledge of what character data were used. The third type of file used by the STK is the taxa substitution file. This file defines which taxa are to be replaced. The substitution can be empty (a removal of that taxon), a single taxon or multiple taxa as a polytomy.

taxon_1 = taxon_2

taxon_3 = taxon_4, taxon_5, taxon_6

taxon_7 =

The above taxa substitution file replaces taxon_1 with taxon_2; taxon_3 with a polytomy of taxon_4, taxon_5 and taxon_6; and removes taxon_7.

#### Perl Module

The Perl module contains over 25 functions used by the executables included in the STK. These provide functionality such as reading and writing NEXUS files, reading and writing XML files, obtaining taxa lists, checking if a taxa is included in a file, etc. These functions are the basis of the executables which form the STK.

#### Executables

There are currently twelve individual application scripts in the STK, each designed to carry out a single step described above or to manipulate the data in some way (Table 1). Below is a description of a few key scripts along with examples of use. For more details see [16].

**Table 1:** Executables in the STK. List of all executables contained in the STK along with a short description

Script Name	Function
Stk_amalagamate_trees	Create a single file from multiple tree files.

Stk_check_data	Provides a consistency check on the current dataset.

Stk_check_overlap	Ensures minimal taxonomic overlap between source trees in the dataset.

Stk_check_substitutions	Checks that a substitution file will not introduce taxa into a dataset. Also checks formatting of a file.

Stk_create_matrix	Create a MRP matrix file from a dataset.

Stk_data_independence	Check the dataset for independence between source trees.

Stk_data_summary	Prints a summary of a dataset, including taxa list and some basic statistics.

Stk_fix_treeview	Converts tree files produced in TreeView [20] to standard NEXUS format.

Stk_replace_genera	Converts a dataset to species-level (as far as possible).

Stk_replace_taxa	Enables substitutions or removal of taxa from a dataset or file.

Stk_search_data	Searches phylogenetic- and meta-data. Can also create a copy from the returned results.

Stk_tree_permutation	Uses a modified NEXUS file to create all permutations of taxa positions for paraphyletic taxa.

##### Check Data

Check Data provides a veracity check for the source data. The script performs three checks, depending on the files that are contained in the dataset; one check examines the NEXUS source phylogenies, the other two checks require the inclusion of XML files. The first check is to ensure that all source phylogenies contained in a dataset are valid NEXUS files as defined by Bio::NEXUS which contains approximately 80% of all known NEXUS commands [14]. The second check ensures the validity of the XML files in the dataset and that they contain a minimal amount of information, e.g. bibliographic information, a taxa list, and the characters used in the study. Third, the XML and source phylogenies are checked to ensure they contain the same taxa.

The check is started with a simple command:

perl stk_check_data.pl --dir ../path/to/dataset

Any errors are displayed on the screen, which in this example is a duplicate taxon in the NEXUS file:

perl stk_check_data.pl --dir ../path/to/dataset

----------------------------------------------------------

File: ../data/example.tre

Duplicate taxon: Margarops_fuscus

##### Check Overlap

**Figure 1:**
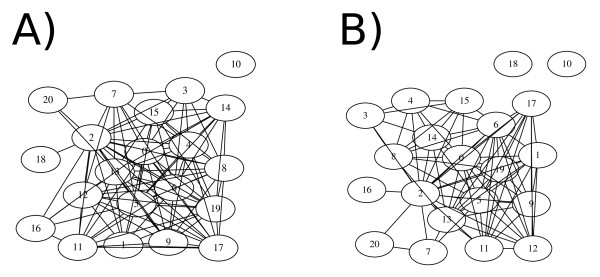
**Checking taxonomic overlap of source phylogenies**. An example of the output from stk_check_overlap. A) shows the source trees represented by nodes with an edge drawn between them when two taxa are shared between them. Tree 10 does not have sufficient taxonomic overlap and should therefore be excluded. B) shows the same set of source phylogenies, but with four taxa required as the minimum for taxonomic overlap. Now trees 10 and 18 should be excluded. In addition, note that the number of edges connecting each node has decreased accordingly.

Check Overlap ensures that there is adequate taxonomic overlap between source phylogenies, in other words all source phylogenies must fulfill the basic requirement for supertree construction of each source (rooted) phylogeny has at least two taxa in common with one other (rooted) source phylogeny [1]. The default options of this executable ensure that a group of source phylogenies fulfill this requirement, but this can be changed to an arbitrary number of taxa, allowing application of more stringent requirements. This script optionally produces a ".dot" file which can be visualised in GraphViz [17]. Example output from GraphViz is shown in figure 1.

The default options, where two taxa must overlap with graphical output (figure 1a):

perl stk_check_data.pl --dir ../path/to/dataset --graphic

To extend to more taxa (figure 1b):

perl stk_check_data.pl --nCluster = 4 --dir ../path/to/dataset -graphic

##### Replace Taxa

This script enables replacement or deletion of taxa. The replacement could be a single taxon or multiple taxa encoded as a polytomy. This replacement can be carried out on a directory or a single file. In addition, the executable can read in a substitution file and carry out multiple replacements or deletions simultaneously.

For example, to remove a taxon from the dataset:

perl stk_replace_taxa.pl --dir ../data --old Xiphocolaptes_promeropirhynchus

To replace a taxa, for example to correct common mis-spellings:

perl stk_replace_taxa.pl --dir ../data --old Gallus_sonnerati --new Gallus_sonneratii

To use a substitution file to carry out multiple replacement and deletions:

perl stk_replace_taxa.pl --dir ../data --taxa subs.txt

where the subs.txt _le is in the format described previously.

##### Search Data

Search Data performs two functions: one is to identify source phylogenies that contain certain data, such as a particular taxon, use of a certain character, etc. Second, the script can create a copy of the data that match the search criteria. In other words, subsets of the dataset can be created easily; e.g. those containing only molecular data.

The following command can be used to create a version of the dataset that contains only those source phylogenies derived from the gene cytochrome b, and place that data in a directory called "new data":

perl stk_search_data.pl --dir ../path/to/data --charterm cytb --copy ../new_data

#### Testing

As part of creating robust software, it is important that the software is extensively tested [18]. The STK contains two automated test suites: a unit test suite and a regression test suite. The unit tests exercises 100% (in terms code statements, branches, and conditionals) of the STK module, checking correct functionality, incorrect inputs are handled correctly and that each function is documented. There are currently over 250 separate tests carried out. The regression tests exercise the scripts, again checking for correct functionality and correct handling of incorrect inputs. Whilst these tests do not ensure bug-free code, they do increase confidence that STK does not contain major errors that would introduce errors into the dataset during processing. Given the number of trees contained in modern datasets, finding and correcting such introduced errors would not be a trivial task.

## Discussion

**Figure 2:**
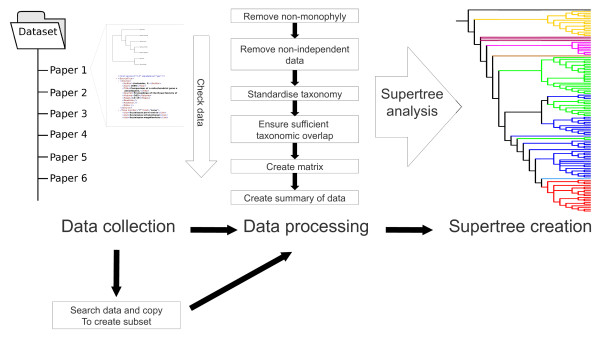
**Chaining of STK scripts to produce a "processing pipeline"**. Data (right - both trees and associated XML meta-data) are processed via the STK scripts. The data output from one script can be fed into another, with data checks being performed throughout processing. The final stage is matrix generation and use of other tools to produce a supertree.

There are a number of software tools to construct and analyse large phylogenies using supertree methods (see [19] for a comprehensive list). However, there is a lack of software available for processing data ready for analysis, as most existing software concentrates on construction or analysis of phylogenies. Given the importance of robust data in supertree construction [2,9], it is important that tools are created to implement processing protocols. However, in order to implement processing protocols meta-data is required in addition to the phylogenetic information. The STK encourages the storage of meta-data and the use of robust protocols for processing data ready for supertree construction. Without a software tool like the STK it is impossible to construct large phylogenies with thousands of taxa following rigorous, robust protocols. We envision the STK to be used as a "processing-pipeline" where data is fed from one script into another, carrying out each step of the protocols as required or desired (figure 2).

### Future work

Although the STK has currently been used successfully in two projects [2,3], there are many useful features that could be implemented. Proposed future features include the addition of a Graphical User Interface to improve the ease-of-use of the STK. We also intend to add revision control which will provide a transparent way of maintaining a full processing log and allow an 'undo' facility to the processing chain.

## Availability and requirements

• **Project name: **Supertree Tool Kit (STK)

• **Project home page: **http://sourceforge.net/projects/stck/

• **Operating system(s): **Platform independent. Tested on Ubuntu and Fedora Linux, Mac OS X, Windows XP.

• **Programming language: **Perl

• **Other requirements: **Perl 5 or higher, and the following Perl modules: XML::Simple, File::Find, File::Spec::Functions, File::Copy, Carp, Bio::NEXUS, Graph, and Graph::Writer::Dot

• **License: **GNU GPL

• **Any restrictions to use by non-academics: **No restrictions

## Competing interests

The authors declare that they have no competing interests.

## Authors' contributions

KED design and conceived the study. JH and KED wrote the code and drafted the manuscript. KED tested the code. All authors read and approved the final manuscript.
